# A novel mutation in *SEPN1* causing rigid spine muscular dystrophy 1: a Case report

**DOI:** 10.1186/s12881-018-0743-1

**Published:** 2019-01-14

**Authors:** Fateme Ziyaee, Eslam Shorafa, Hassan Dastsooz, Parham Habibzadeh, Hamid Nemati, Amir Saeed, Mohammad Silawi, Mohammad Ali Farazi Fard, Mohammad Ali Faghihi, Seyed Alireza Dastgheib

**Affiliations:** 1 Department of Pediatrics, Shiraz University of Medical Sciences, Shiraz, Iran; 2 Italian Institute for Genomic Medicine (IIGM), University of Turin, Turin, Italy; 3 Persian BayanGene Research and Training Center, Dr. Faghihi’s Medical Genetic Center, Shiraz, Iran; 4 Student Research Committee, Shiraz University of Medical Sciences, Shiraz, Iran; 5 Shiraz Neuroscience Research Center, Shiraz University of Medical Sciences, Shiraz, Iran; 6 Department of Psychiatry and Behavioral Sciences, University of Miami Miller School of Medicine, Miami, USA; 7 Department of Genetic, Shiraz University of Medical Sciences, Shiraz, Iran

**Keywords:** Novel mutation, *SEPN1*, Rigid spine muscular dystrophy, Muscular dystrophies, Selenoproteins

## Abstract

**Background:**

Muscular dystrophies are a clinically and genetically heterogeneous group of disorders characterized by variable degrees of progressive muscle degeneration and weakness. There is a wide variability in the age of onset, symptoms and rate of progression in subtypes of these disorders. Herein, we present the results of our study conducted to identify the pathogenic genetic variation involved in our patient affected by rigid spine muscular dystrophy.

**Case presentation:**

A 14-year-old boy, product of a first-cousin marriage, was enrolled in our study with failure to thrive, fatigue, muscular dystrophy, generalized muscular atrophy, kyphoscoliosis, and flexion contracture of the knees and elbows. Whole-exome sequencing (WES) was carried out on the DNA of the patient to investigate all coding regions and uncovered a novel, homozygous missense mutation in *SEPN1* gene (c. 1379 C > T, p.Ser460Phe). This mutation has not been reported before in different public variant databases and also our database (BayanGene), so it is classified as a variation of unknown significance (VUS). Subsequently, it was confirmed that the novel variation was homozygous in our patient and heterozygous in his parents. Different bioinformatics tools showed the damaging effects of the variant on protein. Multiple sequence alignment using BLASTP on ExPASy and WebLogo, revealed the conservation of the mutated residue.

**Conclusion:**

We reported a novel homozygous mutation in *SEPN1* gene that expands our understanding of rigid spine muscular dystrophy. Although bioinformatics analyses of results were in favor of the pathogenicity of the mutation, functional studies are needed to establish the pathogenicity of the variant.

## Background

Muscular dystrophies are a group of disorders with heterogeneous clinical, genetic, and biochemical presentation. They are usually recognized by variable degrees of progressive muscle degeneration and weakness affecting limb, axial, and facial muscles. In some types of these disorders, muscles of the respiratory system and heart, as well as the swallowing process can be involved. Rarely, other tissues and organs, including brain, inner ear, eyes, or skin are also affected. There is a wide variability in the age of onset, symptoms and rate of progression in different forms of these disorders [1–3].

During the past decade, muscular dystrophies have extensively been studied. These advancements have been largely due to the breakthroughs developed in molecular genetics techniques, which have paved the way for the identification of the genetic and molecular basis of many of these disorders, improvements in the standards of care, and novel treatment approaches. Currently, performing molecular genetic diagnosis is very useful for establishment of phenotype-genotype correlations, pre-marital genetic counseling, prenatal diagnosis, and disease prognosis as well as identification of new treatments for these disorders [4–9].

Since identification of the exact function of genes involved in muscular dystrophies, as well as the pathological mechanisms and phenotypic consequences of mutations may shed light on therapeutic strategies for these disorders, the objective of our study was to find the genetic cause of muscular dystrophy in our patient and report the associated observed clinical presentations.

## Case presentation

**Fig. 1:**
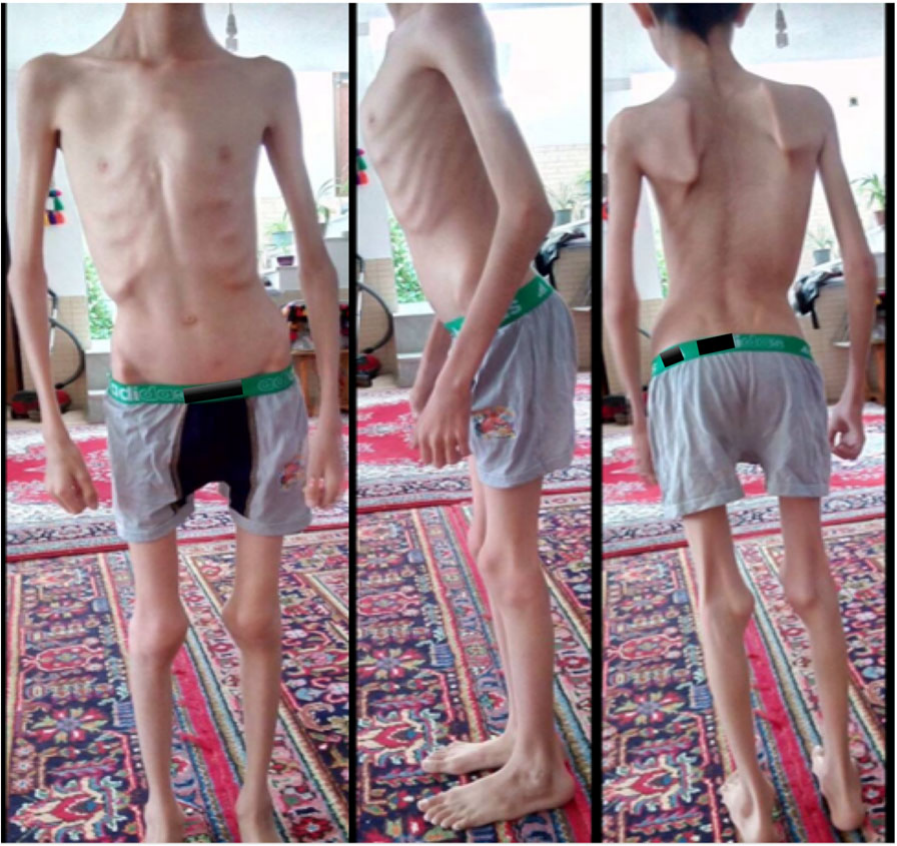
Generalized muscular atrophy, kyphoscoliosis, and flexion contracture of the knees and elbows in our patient

A 14-year-old boy (height = 140 cm, weight = 18 kg) from Fars province, southern Iran, who was born to first-cousin parents without family history of any genetic disorders, was referred to our center with failure to thrive, fatigue, muscular dystrophy, generalized muscular atrophy, kyphoscoliosis, and flexion contracture of the knees and elbows (Fig. 1). His motor symptoms started at the age of four years with frequent episodes of falling down that had progressed in subsequent years. There were no other family members with similar signs or symptoms. He walked at the age of 11 months and had no motor milestone delay. He had two previous admissions to the pediatric intensive care unit due to pneumonia and respiratory distress. The patient had nasal speech and sleep apnea and was under treatment with BiPAP breathing machine. By the age of 12, he was noted to have scoliosis requiring bracing.

On physical examination, the patient was cachectic with generalized muscular atrophy. Decreased muscle power in the shoulder-girdle muscles, foot extensors and limb muscles (4/5 MRC muscle scale) was noted. He also had *pes cavus* and contracture of both knees and elbows. He was also found to have severe spine rigidity with a chin-sternum distance of 15 cm.

Transthoracic echocardiography was only notable for mild pulmonary hypertension and mild tricuspid regurgitation. Pulmonary function testing revealed a FEV_1_ of 35% and FVC of 32% of the predicted values.

Serum calcium and phosphorus levels were 8.2 and 3.1 mg/dL, respectively. The patient had abnormally high levels of creatine phosphokinase (CPK) (340 U/L) and lactate dehydrogenase (LDH) (1200 U/L).

Nerve conduction study was normal. However, needle electromyography (EMG) examination revealed myopathic changes in *deltoid*, *biceps*, *tibialis anterior*, and *rectus femoris* muscles, in favor of Emery-Dreifuss muscular dystrophy.

To identify the mutated gene involved, whole-exome sequencing (WES) was used on genomic DNA extracted from EDTA blood of the patient. Next generation sequencing (NGS) was carried out on an Illumina NextSeq 500 platform to investigate all coding regions and their boundaries. WES details of coverage and number of reads are provided in Table 1. NGS data were analyzed using different bioinformatics tools and databases [10]. NGS data identified a novel, homozygous missense mutation in *SEPN1* gene (chr1:25812784, NM_020451.2: exon:10, c. 1379 C > T, p.Ser460Phe). This mutation has not been reported before in different public variant databases and also our database (BayanGene), so it is classified as a variation of unknown significance (VUS). To confirm the novel mutation identified in our patient, Sanger sequencing was performed using primers covering the mutated exon as follows:

**Table 1:** Whole Exome Sequencing detail of coverage and number of reads

Type	Value	Type	Value
Total Reads	11,709,761	Percent reads on target	95.70%
Passed filter Unique Reads aligned	11,648,030	Percent Passed filter Unique Reads aligned	99.77%
Mean Target Coverage	85X	Percent on Target	92.01%
Percent Duplicate	10.94%	Duplicate in analysis	0%
Capture Method	Agilent Inherited Disease	Total Genes Covered	3204
Run method	NextSeq 500	Sequence length	151 Pair-End
Phred Quality Score above 38	90%	GC content	55%
Nucleotide Covered GTE_1	100%	Nucleotide Covered GTE_5	99%
Nucleotide Covered GTE_8	98%	Nucleotide Covered GTE_10	97%
Nucleotide Covered GTE_15	91%	Nucleotide Covered GTE_20	83%
Nucleotide Covered GTE_30	69%	Nucleotide Covered GTE_40	56%
Nucleotide Covered GTE_50	44%	Nucleotide Covered GTE_60	35%
Nucleotide Covered GTE_70	27%	Nucleotide Covered GTE_80	21%
Nucleotide Covered GTE_90	16%	Nucleotide Covered GTE_100	13%

*GTE* Greater or equal to #

F-SELE: 5’-GCACACACTACAGACTCAGC-3′ and.

R-SELE: 5’-GGAAGACACTTGGTCAAGGTTAC-3′ (443 bp).

**Fig. 2:**
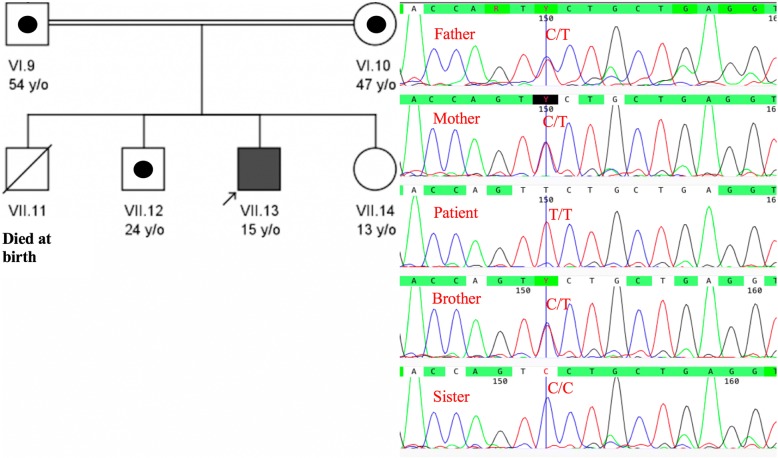
Pedigree and Sanger sequencing chromatogram of family involved in this study

Sanger sequencing confirmed the identified mutation in *SEPN1* in proband as homozygous (T/T). His mother, father and brother were confirmed to be heterozygous (C/T), and his sister homozygous for the wildtype allele (C/C) (Fig. 2).

In order to predict the conservation of the mutated residue, we used different bioinformatics tools, including the Basic Local Alignment Search Tool (BLAST BLASTP ver 2.2.31+) on ExPASy (available from: https://web.expasy.org/cgi-bin/blast/BLAST.pl) and WebLogo (https://weblogo.berkeley.edu/logo.cgi).

**Fig. 3:**
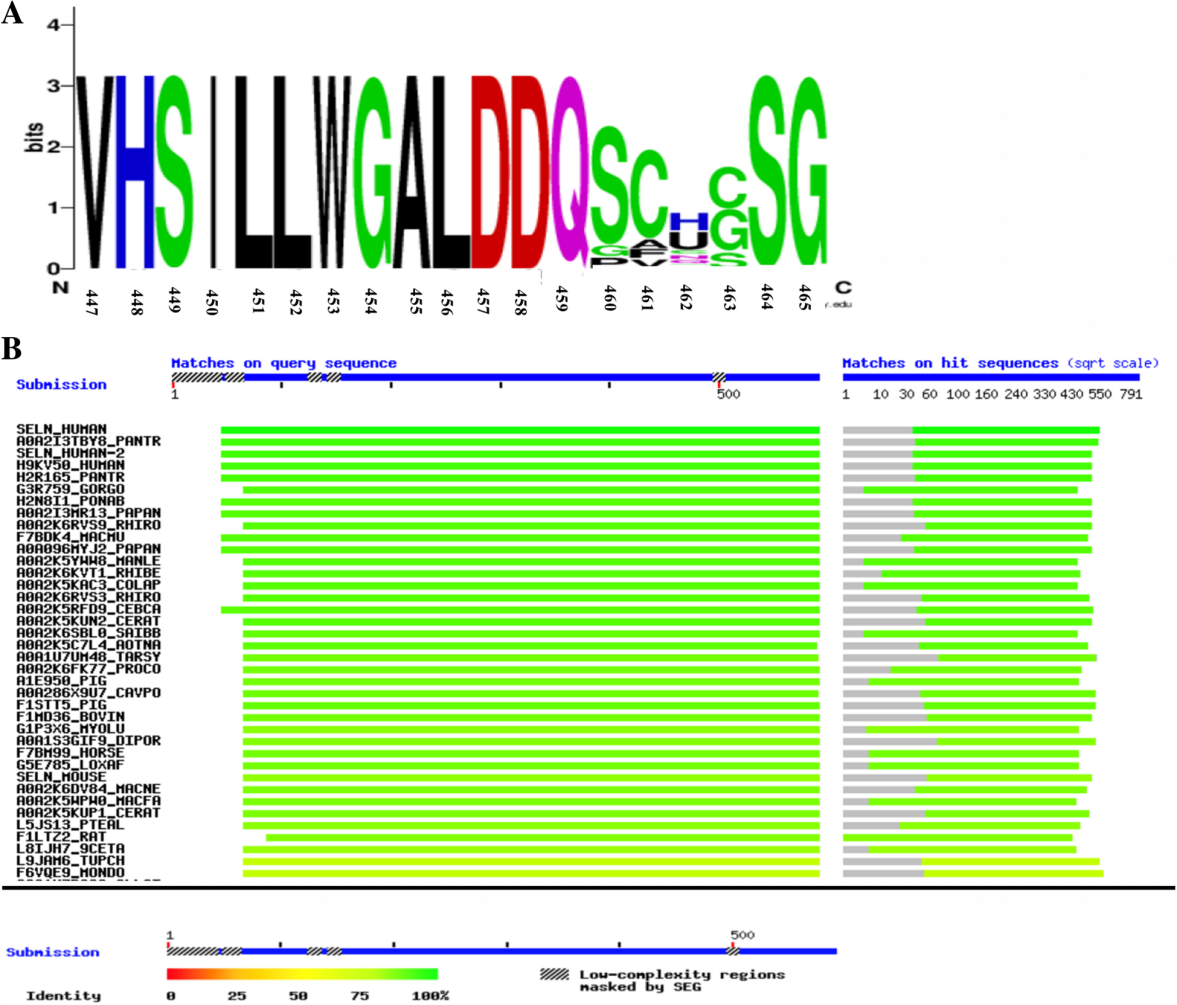
**a**: Graphical representation of amino acid multiple sequence alignment among different species. It shows degree of conservation for amino acids 447–465 of SEPN1 protein. Position 460 (serine) indicates the mutated residue in our study. It has middle conservation degree (2 bits) and also shows that it is replaced by two other amino acids, i.e., glycine and proline in other species but not by phenylalanine. A logo represents the height of each letter proportional to the observed frequency of the corresponding amino acid. The overall height of each stack is proportional to the sequence conservation, measured in bits, at that position. The maximum sequence conservation per site is log_2_ 20 ≈ 4.32 bits for proteins. The protein sequence alignment was performed for the following species: *Homo sapiens* (Q9NZV5), *Gorilla gorilla gorilla* (G3R759), *Pongo abelii* (H2N8I1), *Papio anubis* (A0A2I3MR13), *Rhinopithecus roxellan* (A0A2K6RVS9), *Sus scrofa* (A1E950), *Bos taurus* (F1MD36), *Myotis lucifugus* (G1P3X6), *Dipodomys ordii* (A0A1S3GIF9), *Equus caballus* (F7BM99), *Loxodonta africana* (G5E785), and *Mus musculus* (D3Z2R5). **b**: Multiple protein sequence alignment of SEPN1 across different kingdoms

Bioinformatics analysis predicted that this mutation is damaging and can affect the proper function of the protein (Table 2). In addition, multiple protein sequence alignment revealed conservation of the most residues of *SEPN1* across different species (Fig. 3a and b). Serine residue is conserved among studied species (Fig. 3a). The frequency of serine in this position is more than that for glycine and proline—only these two amino acids replace serine in other species. Since phenylalanine residue is not among these frequent amino acids, the replacement of serine with phenylalanine is predicted to be deleterious.

**Table 2:** Results of bioinformatics analyses of the novel mutation in this study

Chr	Start	Ref	Alt	Function	Gene	BayanGene	avsnp147	Frequency
1	25,812,784	C	T	exonic	SELENON	1	rs767530943	0.000018
SIFT	Polyphen2	LRT	Mutation Taster	Mutation Assessor	FATHMM	REVEL	MCAP	CADD _phred
D	D	N	D	M	D	0.73	.	18.91

## Discussion and conclusion

Desmin-related myopathies (DRM) are a group of muscular disorders with heterogeneous clinical presentation and genetic basis, characterized by intrasarcoplasmic desmin aggregation. It has been found that around 30% of DRM are resulted from impaired desmin gene. The impairment in the structure and subsequent accumulation of desmin result in spinal rigidity, limitation of neck and trunk flexion, progressive scoliosis, early notable limited flexion of the lumbar and cervical spine, which ultimately lead to loss of the spine and thoracic cage movements [11].

Rigid spine muscular dystrophy-1 and myopathy, congenital, with fiber-type disproportion (CFTD) result from disease-causing mutations in *SEPN1* gene (606210), located on chromosome 1p36, which encodes selenoprotein N, a glycoprotein found within the endoplasmic reticulum (ER) [12].

Selenoproteins, which contain selenocysteine residue, are vital for a wide range of biological pathways [13, 14]. Twenty-five selenoprotein-encoding genes have been identified in human genome [15]. The *SEPN1* contains a single selenocysteine residue and has an ER-addressing and -retention signal, indicating its localization within the ER [16, 17]. High level of expression of SEPN1 has been found in several human fetal tissues, including muscles. However, the level of expression is lower in adult tissues, indicating its key role during early stages of embryogenesis, in early development and in cell proliferation [17, 18]. *SEPN1* has a Ca^2+^-binding domain that is involved in the biochemical processes regulating the release of intracellular calcium. This protein is essentially involved in oxidation and reduction reactions, mainly on calcium pumps, modifying the regulation of calcium in ER [19]. Calcium homeostasis is crucial for normal development and differentiation of muscle. Therefore, SEPN1 protein is a vital component in the process of muscle fiber formation and fiber specification [20].

In fact, supporting evidence suggests that selenium, among all its biological roles, is also influential on the normal physiologic state of striated muscles. For instance, selenium deficiency leads to acquired cardiomyopathy [21] and white muscle disease [22]. Likewise, the *SEPN1* mutations are associated with muscular dystrophies.

So far, four autosomal-recessive neuromuscular disorders, collectively regarded as *SEPN1*-related myopathies (SEPN-RM), have been identified [23]. Those include rigid spine muscular dystrophy (RSMD1) [24, 25], the classical form of multiminicore disease (MmD) [23], desmin-related myopathy with Mallory-body like inclusions (MB-DRM) [26], and CFTD [27]. Patients with SEPN-RM present with similar clinical findings, mainly early-onset hypotonia and muscular atrophy, particularly in axial musculature, along with ensuing scoliosis, neck weakness, and spinal rigidity. In patients with impaired respiratory ventilation, fatal prognosis is also expected [28]. However, the disease onset, clinical course and outcome of patients can be very variable [29].

The first *SEPN1* mutation related to a human genetic disorder was found in 2001 in patients affected by congenital RSMD [24]. Over the recent years, various pathogenic and non-pathogenic variations have been identified across *SEPN1* [30, 31]. The initial approach for a reliable clinical diagnosis of these disorders is doing a magnetic resonance imaging (MRI) of muscles, and measuring the growth hormone level, metabolic and muscular serum markers, and performing electrodiagnostic studies and echocardiography [32–38].

In conclusion, we identified a novel *SENP1* mutation, which is predicted to be deleterious due to high damaging scores extracted from various bioinformatics software, conservation of amino acid in studied position, confirmation of mutation in the family, and absence of the mutation in our databases (1000 Iranian Genome).

## Abbreviations

CFTD: Congenital fiber-type disproportion
CPK: Creatine phosphokinase
DRM: Desmin-related myopathies
EMG: Electromyography
ER: Endoplasmic reticulum
LDH: Lactate dehydrogenase
MB-DRM: Desmin-related myopathy with Mallory-body like inclusions
MmD: Multiminicore disease
MRI: Magnetic Resonance Imaging
NGS: Next generation sequencing
RSMD1: Rigid spine muscular dystrophy
SEPN-RM: SEPN1-related myopathies
VUS: Variation of unknown significance
WES: Whole exome sequencing
